# The impact of genetic manipulation of laminin and integrins at the blood–brain barrier

**DOI:** 10.1186/s12987-022-00346-8

**Published:** 2022-06-11

**Authors:** Sebok K. Halder, Arjun Sapkota, Richard Milner

**Affiliations:** San Diego Biomedical Research Institute, 3525 John Hopkins Court, Suite 200, San Diego, CA 92121 USA

**Keywords:** Laminin, Fibronectin, Integrins, Blood vessels, Inflammation, Blood–brain barrier integrity, Angiogenesis

## Abstract

Blood vessels in the central nervous system (CNS) are unique in having high electrical resistance and low permeability, which creates a selective barrier protecting sensitive neural cells within the CNS from potentially harmful components in the blood. The molecular basis of this blood–brain barrier (BBB) is found at the level of endothelial adherens and tight junction protein complexes, extracellular matrix (ECM) components of the vascular basement membrane (BM), and the influence of adjacent pericytes and astrocyte endfeet. Current evidence supports the concept that instructive cues from the BBB ECM are not only important for the development and maturation of CNS blood vessels, but they are also essential for the maintenance of vascular stability and BBB integrity. In this review, we examine the contributions of one of the most abundant ECM proteins, laminin to BBB integrity, and summarize how genetic deletions of different laminin isoforms or their integrin receptors impact BBB development, maturation, and stability.

## Introduction

For cells in certain types of tissue, a basement membrane (BM) is an essential requirement because it directs cell organization within the tissue, as well as imparting strong tissue integrity [1–3]. For this reason, it comes as no surprise that cell types exposed to high levels of physical shear stress, such as the skin epidermis, the gut epithelium and blood vessels, all closely attach to a BM. These structures form early in life as an integral part of the developmental program and disruption of any of the key components of BMs result in catastrophic failure of embryogenesis, thus highlighting the essential role of these structures [4–6]. Blood vessel development and maturation absolutely requires the presence of a BM and absence of one of the abundant BM proteins, laminin, results in failure of vasculogenesis and embryonic lethality [6].

Blood vessels in different organs exhibit different levels of vascular integrity according to local functional requirements. For instance, blood vessels in the glomerulus of the Bowman’s capsule in the kidney have a fenestrated, relatively leaky phenotype to allow the blood contents of the afferent arterioles to pass easily into the renal tubule before being selectively reabsorbed at different stages of the nephron [7]. By contrast, blood vessels in the CNS are very specialized in exhibiting very high levels of vascular integrity and low levels of permeability [8–10]. In this manner, CNS blood vessels carefully regulate the passage of blood-borne agents into the CNS, thus protecting the delicate CNS neural cells from any harmful agents present in blood. This property of CNS blood vessels is known as the blood–brain barrier (BBB).

The BM of CNS blood vessels is a composite of several extracellular matrix (ECM) proteins, the most abundant of which are the laminins, collagen IV, fibronectin and heparan sulphate proteoglycan (HSPG) [8–10]. Laminin has attracted a lot of interest because it is expressed at very high levels in the vascular BM and because in many different cell types, it promotes differentiation and stabilization of cellular behavior [11–15]. In addition, laminin expression within cerebral blood vessels is dynamically altered in many different physiological and pathological conditions. For instance, in ischemic stroke, vascular BM laminin expression is reduced, commensurate with loss of BBB integrity [16–19], but in sub-clinical exposure to chronic mild hypoxia (CMH), laminin expression is actually increased, which correlates with enhanced endothelial tight junction protein expression [20–23]. These observations suggest that by understanding more about the relationship between laminin expression and BBB integrity, we might be better placed to positively impact BBB integrity when it could be clinically beneficial. The purpose of this review is to describe what is known about the outcome of genetic deletion of the different isoforms of laminin and their integrin cell surface receptors on BBB structure and function, and then raise some outstanding questions that need to be addressed in this field.

## The blood–brain barrier

**Fig. 1:**
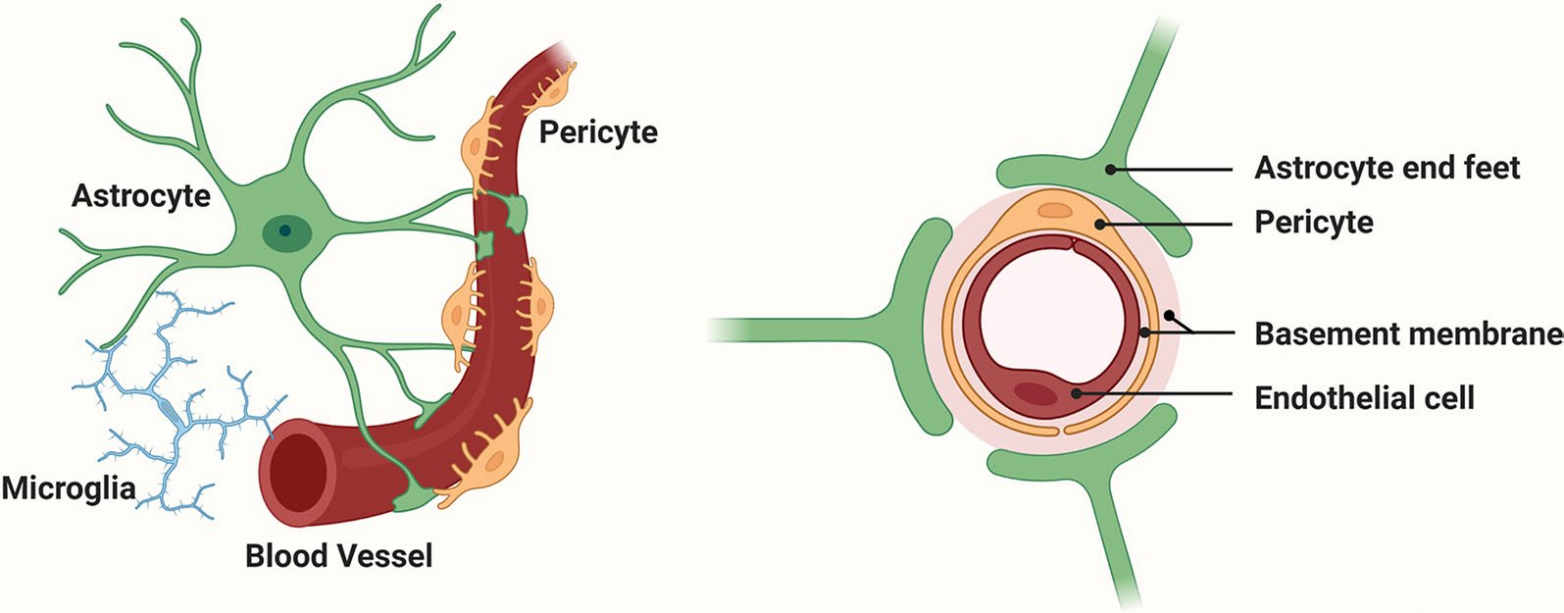
The cellular composition of the blood–brain barrier (BBB). The barrier is formed by a continuum of endothelial cells which line the interior of blood vessels (brown), and which are firmly attached to a basement membrane (BM) composed of a mix of extracellular matrix (ECM) proteins (pink). Pericytes (yellow) are located within the vascular BM. Astrocyte (green) endfeet contact the vascular BM, thus connecting blood vessels to neurons within the brain parenchyma. Microglia (blue) are also in close contact with astrocytes and blood vessels

Compared to blood vessels in other organs, those in the CNS are unique in having high electrical resistance and low permeability, which protects sensitive neural cells in the brain parenchyma from the potentially harmful impact of blood components [8, 9, 24–27]. This low permeability of CNS blood vessels is referred to as the BBB, which is composed of several different cell types, including endothelial cells, pericytes, and the endfeet of astrocytes (Fig. 1). Recent studies have also highlighted the role of adjacent microglia in contributing to a tighter BBB under different challenging stimuli [28–31]. The BBB occurs at the level of CNS capillaries, which comprise endothelial cells attached to a vascular BM composed of different ECM proteins [24, 25, 32]. Pericytes form an integral part of these capillaries and are in close contact with endothelial cells and the vascular BM [33–35]. In addition, a vast network of astrocyte endfeet originating from within the brain parenchyma contact the vascular BM [8, 36–38]. What makes CNS blood vessels so much tighter (50–100-fold greater) than blood vessels in peripheral organs? We now know that three main types of molecular mechanism account for these properties. First, the highly organized expression of endothelial tight junction and adherens proteins make exceedingly tight connections between adjacent endothelial cells. These tight junction proteins (TJPs) include claudins, occludin, and junctional adhesion molecules (JAMs), which attach to the cellular actin cytoskeleton via zonula occludens proteins such as ZO-1 [39–41]. Adherens proteins such as VE-cadherin further act to strengthen the bonds between neighboring endothelial cells [42, 43]. Second, astrocytes and pericytes positively impact BBB integrity. The influence of astrocytes to increases the tightness of blood vessels has been known for many years since the seminal work of Janzer and Raff [37], but more recently, the role of pericytes has gained more attention [33–35]. Third, and sometimes underestimated, is the impact of the vascular BM to which the endothelial cells and other cells attach [36].

The BBB is disrupted in many different neurological conditions, including meningitis, ischemic stroke, multiple sclerosis, and CNS tumors [44–48]. Accumulating evidence suggest that it also deteriorates as part of the aging process and may be an important contributory factor in the pathogenesis of vascular dementia by triggering neuronal dysfunction and neurodegeneration [49–53]. Further studies have shown that BBB integrity is also transiently disrupted by low oxygen levels (hypoxia) [29, 54, 55]. It is interesting that several of these neurological conditions are accompanied by alterations in ECM components of the vascular BM [18, 56–58], raising the possibility that disordered expression of ECM proteins may itself trigger altered behavior in endothelial cells, resulting in reduced BBB integrity. Based on this knowledge, it becomes clear that if we can identify factors that positively regulate BBB integrity, we might be able to therapeutically delay or attenuate the pathogenesis of many neurological diseases.

## Laminins and other vascular basement membrane extracellular matrix proteins

The vascular BM is composed of several different ECM proteins, of which the laminins, collagen IV, fibronectin and heparan sulphate proteoglycan (HSPG) are the most abundant [59–63]. Endothelial cells form strong adhesive attachments to the BM but it must be remembered that this is not just an adhesive carpet which the endothelial cells attach to, but its components also provide important instructive cues that regulate many aspects of cell behavior, including cell adhesion, survival, proliferation, migration and differentiation [60, 64]. Cells bind to ECM proteins primarily via cell surface receptors called integrins and some also use dystroglycan [65–69].

### Different laminin isoforms

**Fig. 2:**
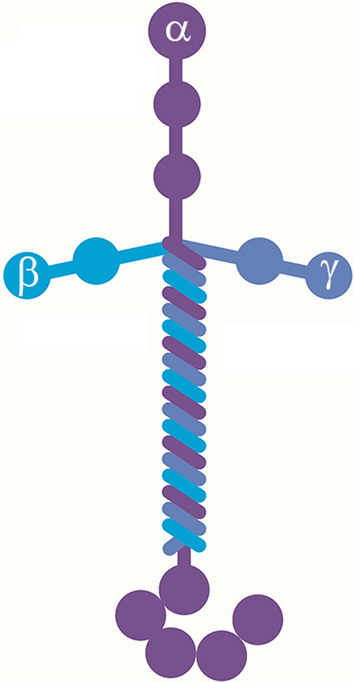
The molecular structure of laminin. Laminins are heterotrimers consisting of α (purple), β, (bright blue) and γ (grey blue) subunits of which 5α, 4β and 3 γ have been so far defined. Laminins are named according to their subunit composition; for instance, laminin-111 comprises the subunits α1, β1 and γ1

One of the most abundant ECM proteins found in the vascular basal lamina is laminin, or more correctly the laminin group of molecules, as there are several different laminin isoforms [61, 62, 70]. Laminins are heterotrimers consisting of α, β and γ subunits (Fig. 2), of which 5 α, 4 β and 3 γ have been so far defined allowing the generation of up to 20 different laminin isoforms [71]. The current nomenclature dictates that laminins are named according to their subunit make-up, so for instance, laminin-111 is composed of the subunits α1, β1 and γ1.

### Differential expression and functions of different laminin isoforms in the CNS

Histological studies of the adult CNS reveal that laminin staining is found exclusively in a vascular pattern, with negligible levels in the tissue parenchyma [72–74]. The contributions to this vascular BM laminin originate from several different cell types, each of which synthesize different laminin isoforms. More than 20 years ago, Sixt et al. performed an elegant immunohistochemical study in an experimental autoimmune encephalomyelitis (EAE) animal model of multiple sclerosis, to show that during infiltration of inflammatory leukocytes, the two layers of the vascular BM (endothelial and parenchymal) of CNS blood vessels get separated by infiltrating leukocytes which congregate in the gap in-between the two layers, in the so-called perivascular space [74]. In this system it was demonstrated that the inner endothelial layer of BM contains laminin-411 and -511, while the parenchymal layer of BM contains laminin-211 synthesized by astrocytes, and laminin-111 produced by leptomeningeal cells. They also noted that while endothelial cells synthesize both laminin-411 and -511, laminin-411 was widely expressed throughout the vascular BM of blood vessels, while expression of the 511 isoform was discontinuous. Interestingly, at sites of inflammatory cell extravasation in the EAE model, it was noted that leukocytes tend to breach the BBB at sites expressing laminin-411 but not in regions expressing the 511 isoform, suggesting permissive and inhibitory functions for these distinct laminin isoforms, respectively. Recent studies have demonstrated that pericytes express the same laminin isoforms as endothelial cells, laminin-411 and -511 [75]. Interestingly, developmental studies revealed that while laminin-411 is detected in capillaries as early as embryonic day 11 (E11) [76], laminin-511 appears much later, around 3–4 weeks after birth [77, 78]. It is also notable that laminin-111, which is produced by leptomeningeal cells, does not surround all types of CNS vessel, but is restricted exclusively to arterioles and venules, and is absent from capillaries [74]. The reason for this is that laminin-111 expression is limited to leptomeningeal fibroblasts within meningeal blood vessels, which early in development are present only on the surface of the brain, but during neurodevelopment these vessels invaginate deep within the brain to make contributions to the arteriolar and venular circulation.

### Deletion of different laminin isoforms present unique phenotypes

As members of the laminin family are composed of three subunits, α, β and γ, the impact of deletion of any one subunit depends largely on how widely that subunit is expressed amongst different tissues and cell types [60–62, 70, 71]. This is well illustrated by global deletion of the γ1 laminin subunit which shows an embryonic lethal phenotype due to total loss of the ability to develop basement membranes and thus blood vessels [6]. Global knockouts of other laminin subunits also impact mortality but tend to affect later stages of development, primarily in the postnatal period [79, 80].

#### Global deletion of the laminin α4 subunit

Global deletion of the α4 subunit, which contributes one of the endothelial laminins (411) leads to disrupted vascular development, comprising vascular BM defects due to reduced synthesis of other ECM components, vessel dilation, disordered angiogenesis and reduced vessel integrity [80]. While laminin α4 mutant show increased mortality, the majority of mice survive, most likely due to compensatory increases in the other endothelial laminin, laminin-511 [81]. As CNS blood vessels in wild type mice show a continuous expression of the α4 subunit throughout the vascular BM, but a patchy discontinuous expression of the α5 subunit [74], Wu et al. exploited the finding that in α4 subunit-deficient mice, the laminin α5 subunit becomes ubiquitously expressed throughout the BM by showing that this correlates with a marked reduction in leukocyte infiltration across the BBB in the inflammatory EAE model [81]. These findings supported previous work from the same lab, that leukocytes tend to breach the BBB at laminin-411 expressing sites but not where laminin-511 is present [74]. This study therefore presents an interesting paradox in that while loss of laminin-411 appear to disrupt vessel stability and integrity early during development, in those mice that survive this period, the compensatory upregulation of laminin-511 in α4 subunit deficient mice actually enhances vessel integrity by reducing leukocyte extravasation.

#### Deletion of the laminin α2 subunit

Mice globally deficient in the laminin α2 subunit exhibit growth retardation and severe muscular dystrophic symptoms and die by 5 weeks of age [82]. In the CNS, the α2 laminin subunit is expressed predominantly by astrocytes [74]. Analysis of laminin α2 KO mice at an earlier (3-week) timepoint showed that they had defective BBB integrity, indicated by enhanced vascular leak of Evans Blue tracer, which correlated with delayed vascular maturation, indicated by enhanced expression of MECA-32, a marker of immature BBB, as well as reduced levels of VE-cadherin and the tight junction protein claudin-5 [79]. Global loss of the laminin α2 subunit also had profound effects on the behavior of two other CNS vascular cell types, astrocytes and pericytes, such that astrocytes in these mice showed hypertrophic endfeet which expressed higher levels of GFAP and lacked polarized aquaporin-4 channels. At the same time, pericyte coverage of blood vessels was also substantially reduced [79].

#### Astrocyte-specific laminin deletion

Chen et al. targeted the role of astrocyte laminins specifically by deleting the laminin γ1 subunit specifically in astrocytes, which effectively removes all laminin expression [83]. This astrocyte-specific knockout mouse strain had a milder phenotype than the global or brain-specific α2 laminin subunit knockout in that they survived beyond the 4-week timepoint, but after 2–3 months of age, transgenic mice presented with spontaneous intracerebral hemorrhage (ICH), which became more pronounced with age, such that by 6 months of age, more than 60% of mice had ICH. Most hemorrhages occurred in small arterioles in deep brain regions including the basal ganglia, thalamus and hypothalamus. Closer analysis revealed that while in wild type mice, astrocyte endfeet co-localized strongly with α-SMA positive smooth muscle cells (SMCs) in small arterioles, in the astrocyte-specific laminin KO strain, the number of α-SMA-positive cells was markedly reduced. Based on this, the authors concluded that lack of astrocyte laminin disrupts the function and proliferation of vascular SMCs, resulting in weakening of the arteriolar wall and eventually vessel rupture.

In a complimentary study, the authors used the same mouse strain to show that astrocyte-specific deletion of laminins resulted in BBB breakdown, most likely as a consequence of altering the behavior of pericytes, from a BBB-stabilizing to BBB-disruptive phenotype [84]. In their model, they suggest that astrocyte laminin promotes the BBB-stabilizing phenotype via signaling through the pericyte α2β1 integrin, but loss of this signaling leads to a contractile BBB-disruptive pericyte phenotype. They also showed that loss of astrocyte laminin led to reduced AQP-4 expression by astrocyte endfeet, as well as reduced levels of endothelial tight junction protein expression. These last two events are consistent with the phenotype of the brain-specific-laminin α2 subunit knockout described above [79].

#### Pericyte-specific laminin deletion

More recently, laminins have also been deleted specifically from pericytes by crossing PDGFRβ-Cre mice with floxed laminin γ1 subunit mice [85]. All transgenic mice typically die by 4 months of age due to a severe muscular dystrophy (MD) phenotype. In addition to the MD phenotype, a small percentage (approximately 11%) of progeny also displayed a hydrocephalus phenotype, which became manifest 2 weeks after birth. Interestingly, all mice displaying hydrocephalus also showed BBB disruption, which was associated with reduced expression of the tight junction protein ZO-1 and vascular AQP-4 expression, in addition to dramatically reduced pericyte coverage. Of note, when these transgenic mice were crossed from a C57BL6-FVB mixed background onto a pure C57BL6 background, they failed to develop hydrocephalus and while BBB integrity was normal at 4 months of age, by 8 months old, mild BBB disruption became apparent [75]. To examine whether this pericyte-specific laminin KO strain shows worse pathology in disease models, the authors compared responses with wild type mice in a collagenase-induced ICH model. This revealed that the KO strain showed greater pathology as shown by increased size of hematoma, worse neurological function, reduced BBB integrity and increased neuronal death [86].

#### Deletion of the laminin α5 subunit specifically in endothelial cells or pericytes reveal opposite effects on BBB integrity

As global knockout of the laminin α5 subunit shows an embryonic lethal phenotype, it has been challenging to study the role of this subunit in BBB regulation [87]. Within the BBB, both endothelial cells [74] and pericytes [85] have been shown to express this laminin subunit, making it important to determine the relative contributions of each cell type’s laminin α5 to BBB stability. To address this question, the Yao lab recently generated distinct transgenic mice strains in which laminin α5 was specifically deleted in endothelial cells [88] or pericytes [89]. In both strains of knockout mice, under homeostatic control conditions, loss of laminin α5 had no obvious effect on BBB integrity. In keeping with other studies demonstrating BBB-enhancing effects of laminins, compared with wild type littermates, the EC-laminin α5-KO strain displayed increased BBB permeability in an ICH model, correlating with greater injury volume, leukocyte intravasation and gliosis [88]. However, in contrast, in an ischemic stroke model, the PC-laminin α5-KO strain showed milder BBB disruption compared to wild type controls, which correlated with reduced infarct volume, reduced leukocyte infiltration and improved neurological score [89]. These findings imply that while endothelial laminin α5 positively contributes to BBB integrity, surprisingly, pericyte laminin α5 appears to have detrimental effects on BBB stability, at least in these separate disease models under these conditions.

When all outcomes of the various laminin knockout strains are studied, some common themes stand out. The most important is that generally speaking, loss of laminin, whether it be global or cell-type specific in any of the BBB cell types examined (endothelial cells, astrocytes or pericytes), all result in reduced levels of BBB integrity [79, 83–86, 88]. In the same vein, reduced vascular integrity in all these knockouts is associated with disrupted vascular BM composition, meaning that loss of laminin also negatively impacts the synthesis of other ECM components within the BM. At the same time, reduced expression of endothelial tight junction proteins is also common, as is disordered expression of astrocyte AQP-4 clustering on astrocyte endfeet, reduced pericyte coverage, and in the case of astrocyte-specific laminin deletion, loss of arteriolar SMCs. In conclusion, deletion of most laminin isoforms has deleterious effects on BBB integrity and function. The two notable exceptions to this rule are first, the PC-laminin α5-KO strain, which displays reduced BBB breakdown in an ischemic stroke model, and second, the global α4 laminin subunit KO, because despite showing reduced vascular integrity and disordered angiogenesis early in life, the work of Wu et al. demonstrates that in global α4 laminin subunit KO mice that survive the postnatal period, loss of expression of the α4 laminin subunit leads to compensatory ubiquitous expression of the laminin α5 subunit on all blood vessels, which closely correlates with reduced infiltration of inflammatory leukocytes in the EAE model [81].

## Integrins

**Fig. 3:**
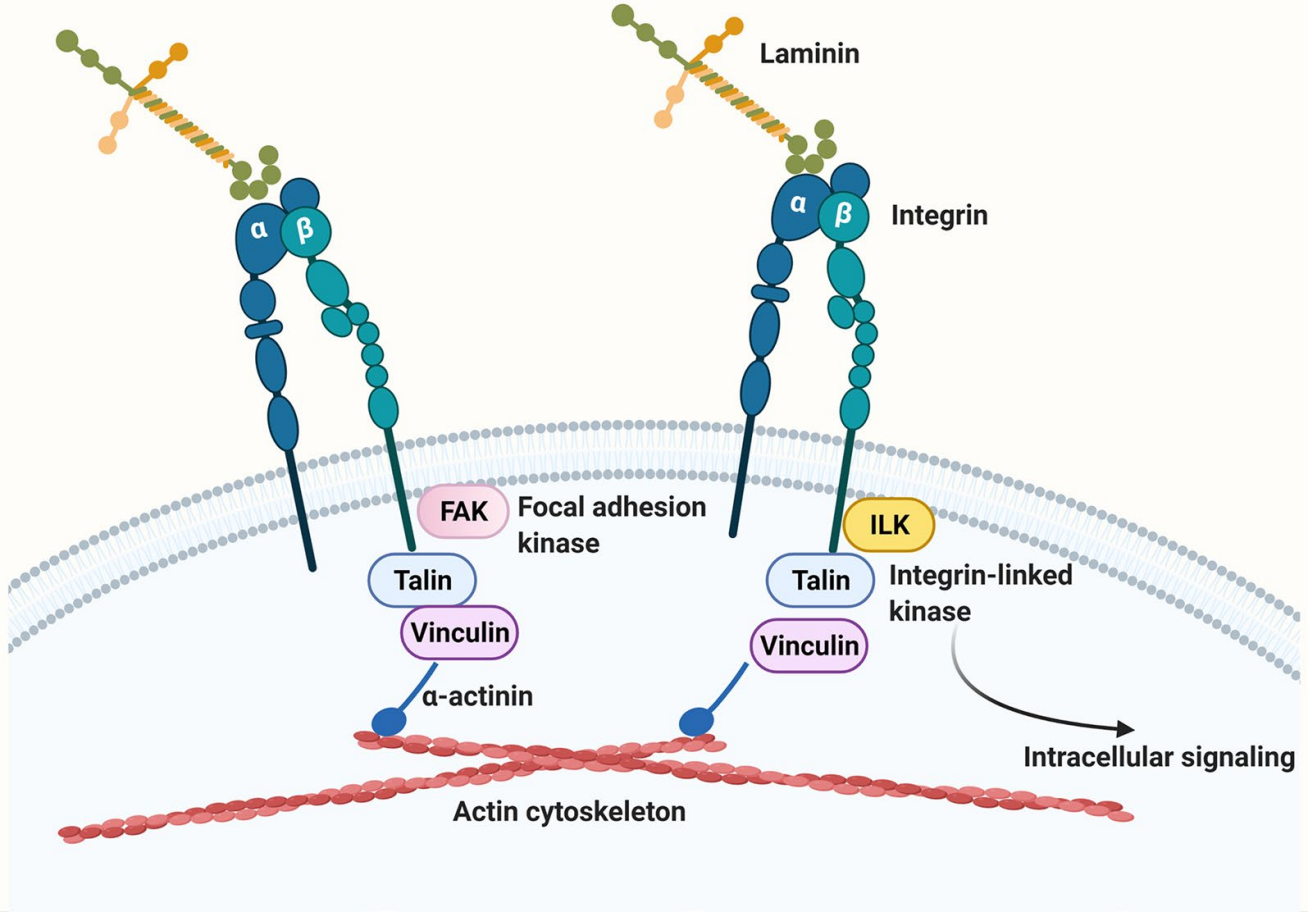
Schematic of laminin-integrin interactions. Laminins in the ECM bind to their cognate cell surface receptors, integrins which are transmembrane proteins consisting of non-covalently linked αβ heterodimers. Integrins perform two vital functions: (i) the cytoplasmic domain of β subunits bind to the cytoplasmic adaptor proteins talin, vinculin and α-actinin to form a transmembrane link between the ECM and the actin cytoskeleton, and (ii) the cytoplasmic domain of β subunits also bind several different cytoplasmic signaling proteins including focal adhesion kinase (FAK) and integrin-linked kinase (ILK) to trigger intracellular signaling cascades

Cells interact with the ECM by way of cell surface receptors called integrins [65, 90]. Once thought of as merely adhesion proteins that cells bind to within tissues, over the last 30 years it has become clear that ECM-integrin interactions play a key instructive role in directing many aspects of cell behavior including cell survival, proliferation, migration, and differentiation [65, 90–93]. Almost all cells in the body express integrins (red blood cells are a rare exception) and the different cell types making up blood vessels in the CNS are no exception. Broadly speaking, integrins achieve their effects by two means (Fig. 3). First, they provide a strong physical connection between the ECM proteins and the actin cytoskeleton. Specifically, the cytoplasmic domains of integrin β subunits interact with cytoplasmic adaptor proteins such as talin, vinculin and α-actinin to form strong bonds with the actin cytoskeleton. Second, by interacting with different cytoplasmic adaptor proteins, they transduce bi-directional signals (outside-in and inside-out) across the cell membrane [65, 91, 94]. In this way, integrin cytoplasmic domains interact with signaling proteins such as focal adhesion kinase (FAK), or integrin-linked kinase (ILK) to stimulate additional intracellular signaling cascades.

### Integrin dimers and ligand specificity

**Fig. 4:**
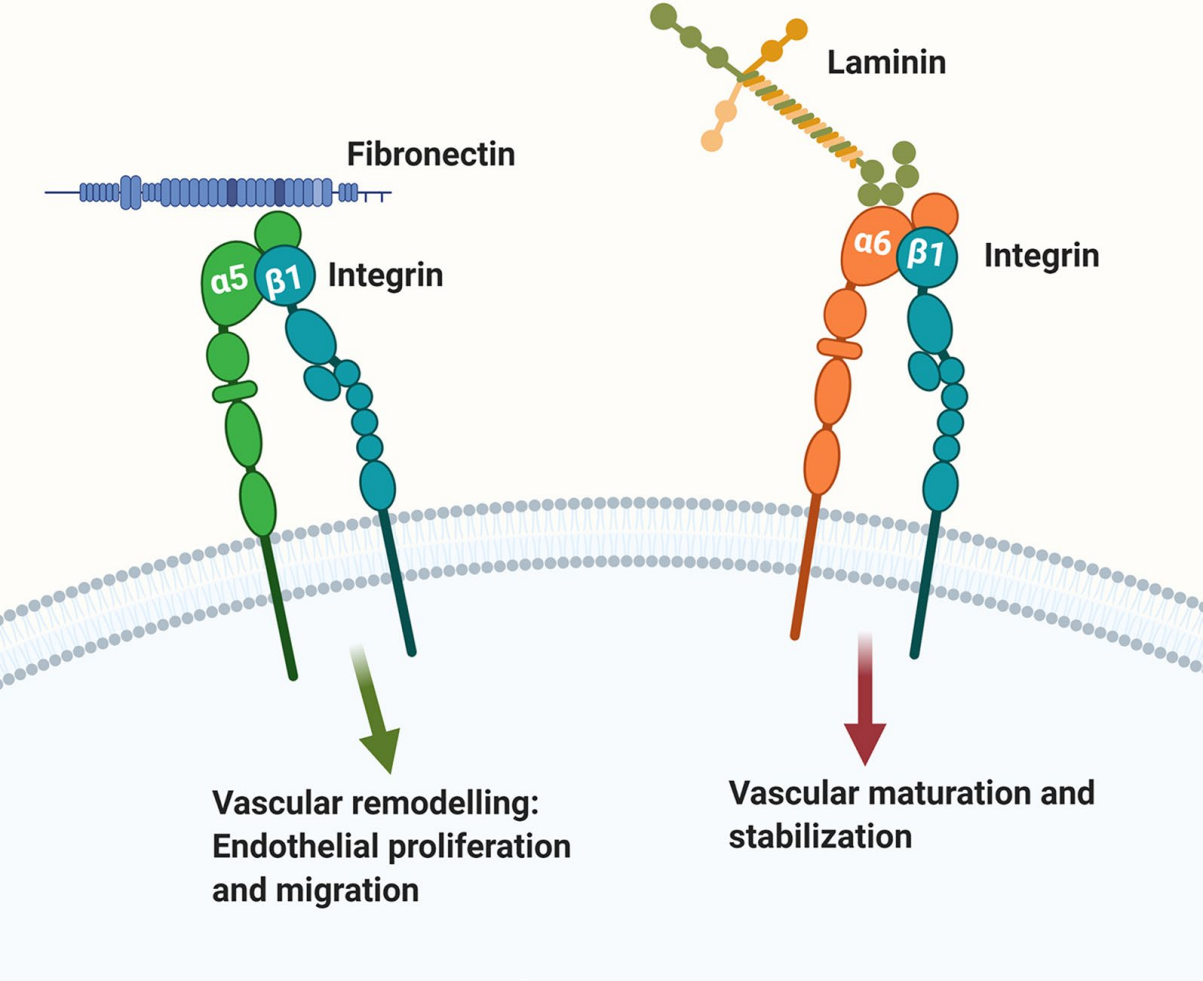
The “green light-red light” model explaining ECM regulation of endothelial cell behavior at the BBB. According to this model, fibronectin-α5β1 integrin and laminin-α6β1 integrin interactions play opposing roles in directing endothelial behavior within CNS blood vessels. Fibronectin-α5 integrin mediated signaling (green arrow) drives vascular remodeling behaviors including endothelial proliferation and migration, while in contrast, laminin-α6β1 integrin mediated signaling (red arrow) promotes endothelial differentiation, resulting in BBB maturation and stabilization

Integrins are a major family of cell adhesion molecules that are expressed as cell surface non-covalently linked αβ heterodimers (Fig. 3) [65, 91, 94]. Currently 16 different α and 8 different β mammalian integrin subunits have been identified, that form up to 24 different integrin heterodimers. The biggest class of integrins is the β1 class which constitutes more than 10 different members that include α1β1, α2β1, α3β1 etc. Each integrin heterodimer has unique ligand specificity. For example, the α5β1 is a fibronectin receptor [94], while the two α6 integrins, α6β1 and α6β4 are both receptors for laminin [95–100] (Fig. 4).

### Differential expression in cerebral blood vessels

It is well established that ECM-integrin interactions play a major role in directing blood vessel formation, maturation, and homeostatic function throughout life. More than 20 years ago, several studies showed that in the adult CNS, β1 integrin expression occurs at the highest levels on blood vessels, with barely detectable signal within the brain parenchyma, suggesting an important role for this class of molecules in the regulation of blood vessel behavior [72, 101–103]. Furthermore, we described a developmental switch in the expression of ECM-integrin proteins during blood vessel development in the CNS [72]. Early in the postnatal period when angiogenesis is ongoing, angiogenic blood vessels express high levels of fibronectin and the fibronectin-binding integrin receptors α5β1 and α4β1 integrins, but maturation of the CNS is accompanied by downregulation of these molecules and upregulation of laminin and the laminin receptor α6β1 integrin as well as the collagen receptor α1β1 integrin. Blood vessels in the normal adult CNS express significant levels of the integrins α1β1 (collagen receptor) and α3β1, α6β1 and α6β4 (laminin receptors) in keeping with laminin and collagen IV being the dominant ECM proteins expressed in the vascular BM [101, 103–105]. By contrast to the other integrins expressed, α6β4 integrin shows an unusual expression pattern on CNS blood vessels, being restricted to arterioles [106]. The exception to this rule is that during acute neuroinflammation such as that seen in the EAE model, α6β4 integrin is induced by other types of blood vessel including capillaries, prompting us to suggest that this induction may be part of a protective adaptive response to enhance BBB integrity in times of extreme insult [107]. In addition, along with several others, we have also shown that during vascular remodeling situations that occur during hypoxia or ischemia, angiogenic CNS blood vessels show marked upregulation of fibronectin and two fibronectin receptors α5β1 and αvβ3 integrins [18, 56, 108, 109].

### Deletion of endothelial integrins reveals different functions

#### α6β4: a unique integrin with several functions

α6β4 is an unusual integrin because in all organs examined including the CNS, its expression is limited to endothelial cells lining arterioles [106]. Endothelial-specific deletion of this integrin resulted in total loss of vascular β4 integrin expression, but β4-EC-KO mice are viable and fertile and show no obvious defects in vascular development or BBB integrity [106]. However, when challenged by chronic mild hypoxia (CMH, 8% O_2_ for periods up to 14 days), in contrast to wild type mice, which mount a strong arteriogenic remodeling response resulting in increased arteriolar density, β4-EC-KO mice showed reduced arteriolar remodeling that correlated with attenuated transforming growth factor (TGF)-β signaling. Of note, in the epidermis, α6β4 integrin plays an essential role in providing structural support to protect skin cells from high shear stress, and deletion of this integrin leads to epithelial detachment in mice [110] and the skin blistering condition junctional epidermolysis bullosa in humans [111, 112]. By comparison, as arterioles are exposed to the highest levels of shear stress, it seems likely that α6β4 integrin is induced at this location in order to confer structural support for the endothelial cells exposed to turbulent forces. In addition to playing this protective role, our observations in β4-EC-KO mice also imply that α6β4 integrin may transduce changes in arteriolar shear stress to facilitate arteriolar remodeling under hypoxic conditions, via interactions with the TGF-β signaling pathway [106].

While in the normal CNS, α6β4 integrin expression is restricted to arterioles [106], in the neuroinflammatory CNS such as that seen in EAE or in transgenic mice overexpressing the pro-inflammatory cytokines interleukin (IL)-6 or interferon-α in the CNS, α6β4 integrin expression is also induced on other types of blood vessels including capillaries [73, 107]. Based on these observations, we wondered if endothelial α6β4 integrin induction within brain capillaries is part of an endogenous protective response to stabilize BBB integrity under inflammatory conditions. This was tested by comparing EAE severity in β4-EC-KO and wild type littermate mice, which showed that β4-EC-KO mice had worse clinical disease that was underpinned by greater levels of BBB breakdown, leukocyte infiltration, and loss of endothelial tight junction protein expression [107]. Thus, our work defines two distinct roles for the endothelial α6β4 integrin in cerebral blood vessels: (i) to promote arteriogenic remodeling under hypoxic conditions, and (ii) to enhance vascular integrity under neuroinflammatory conditions.

#### Reduced expression of endothelial β1 integrins leads to increased BBB leak

It is technically challenging to examine the impact of β1 integrin deletion at the BBB because β1 integrin deletion in endothelial cells results in embryonic lethality, highlighting the essential role of these molecules in vascular development [113, 114]. However, using an inducible Cre-Lox approach, the del Zoppo lab showed that 50% reduction in β1 integrin expression on cerebral vessels led to commensurate decreases in vascular expression of the cognate ECM ligands laminin and collagen IV as well as marked decrease in BBB integrity as indicated by increased IgG leakage [115]. This work supported previous findings from the same group showing that intra-cerebral injection of function-blocking anti-β1 integrin antibodies resulted in greater vascular cerebrovascular leak [116]. Taken together, these studies highlight an important BBB stabilizing role for β1 integrins in CNS blood vessels.

#### α5β1 integrin drives cerebral angiogenesis and lack of this integrin leads to delayed vascular remodeling

Building on our observation that cerebral endothelial cells show a developmental switch in their use of β1 integrins, from α5β1 and α4β1 during developmental angiogenesis to α1β1 and α6β1 in mature blood vessels [72], we went on to demonstrate that angiogenic vessels in the adult CNS also strongly upregulate fibronectin and the α5β1 integrin [18, 23, 117, 118]. As the fibronectin-α5β1 integrin axis has been shown to be important for driving angiogenesis in tumors [119, 120], we then tested in the chronic mild hypoxia (CMH) model whether deletion of endothelial α5β1 integrin impacts hypoxia-driven angiogenesis. This showed that α5-EC-KO mice displayed an attenuated angiogenic response which correlated with delayed endothelial proliferation and CNS vascularization [121].

As CNS blood vessels also upregulate fibronectin and the α5β1 integrin under inflammatory conditions [117], in a separate project we evaluated how absence of α5β1 integrin impacts vascular remodeling and integrity, and clinical disease in the EAE neuroinflammatory model. Interestingly, this revealed that α5-EC-KO mice showed earlier onset and faster progression of clinical EAE disease, though with time, peak disease and chronic disease severity were no different from wild type littermates [122]. Concordant with the accelerated clinical disease, at this early stage of EAE progression, α5-EC-KO mice displayed greater BBB breakdown and enhanced leukocyte infiltration. Consistent with our previous findings [121], α5-EC-KO mice also showed reduced endothelial proliferation, culminating in reduced levels of vascularity as compared with wild type littermates. These findings suggest that α5β1 integrin-mediated vascular remodeling represents an important vascular repair mechanism that counterbalances vascular disruption at an early stage of EAE development.

In a different disease model, the Bix lab recently reported that α5-EC-KO mice are highly resistant to experimental ischemic stroke, displaying smaller ischemic infarcts and reduced levels of BBB breakdown compared to wild type littermates [48]. This suggests that in this model, absence of α5β1 integrin leads to more stable blood vessels that are less likely to undergo vascular remodeling and thus transient BBB disruption. So how can absence of endothelial α5β1 integrin be harmful in EAE but protective in ischemic stroke? We believe that the answer may lie in the fact that stroke is a much more acute and severe insult, while in EAE, the insult is mild and more chronic. In stroke, the vascular disruption is so fast and severe that any repair that α5β1 integrin promotes is quickly overwhelmed, and so silencing α5β1 integrin function acts to ameliorate the vascular breakdown process. By contrast, the milder, more chronic vascular challenge seen in EAE can be opposed by the vascular remodeling events promoted by α5β1 integrin, resulting in faster vascular repair and vessel stabilization. Interestingly, recent studies have shown that endothelial α5β1 integrin is also upregulated on cerebral blood vessels in the bilateral carotid artery stenosis (BCAS) animal model of vascular dementia, raising the possibility that deletion or inhibition of this integrin may help to stabilize BBB integrity and protect against neurological sequelae [57], as demonstrated in the stroke model [48].

## Conclusions and future directions

An intact BBB is an essential prerequisite for the maintenance of good cerebral health, and yet BBB disruption occurs during many neurological conditions [44–48]. Recent evidence indicates it also slowly deteriorates as part of the normal aging process [49–53]. Overwhelming studies support the concept that ECM-integrin interactions are not only important for the development and maturation of cerebral blood vessels; they are also essential for the maintenance of vascular stability and BBB integrity under normal conditions. It is no coincidence that the laminin family of molecules are expressed at such high levels in the vascular BM of CNS blood vessels because genetic deletions of specific laminin isoforms in specific BBB cell types all confirm the importance of this class of molecules in conferring BBB properties in the endothelial cells, pericytes and astrocytes making up the BBB. Indeed, most laminin KOs show common phenotypes that include reduced vascular integrity, disrupted vascular BM composition of other ECM components, reduced expression of endothelial tight junction proteins, disordered expression of astrocyte AQP-4 clustering on astrocyte endfeet and reduced pericyte coverage [79, 83–86, 88]. While genetic deletion studies examining roles of specific integrins at the BBB currently lag behind the laminin field, recent work has demonstrated the importance of the β1 class of integrins in maintaining a tight BBB [115], as well as a key role for α5β1 integrin in promoting endothelial proliferation and cerebral angiogenesis under mild hypoxia and chronic inflammatory disease conditions [121, 122]. In addition, genetic studies have defined two separate roles for the α6β4 integrin both in promoting arteriogenesis and in conferring enhanced BBB stability under chronic inflammatory conditions [106, 107]. Based on current evidence, we propose a simple “green light-red light” model to explain how the fibronectin-α5β1 integrin and laminin-α6β1 integrin axes play important but opposing roles in directing endothelial behavior within CNS blood vessels (Fig. 4). In this model, fibronectin via its interactions with α5β1 integrin (green arrow) drives endothelial proliferation and migration during vascular remodeling scenarios including hypoxia, ischemia and inflammation. By contrast, the laminins, via interactions with α6β1 integrin (red arrow), promote endothelial differentiation, resulting in maturation and stabilization of the BBB.

In future studies it will be interesting to address several outstanding questions. First, as both α4 and α5 laminin subunits are expressed by endothelial cells and pericytes [74, 85], and deletion of the laminin α5 subunit seems to have opposite effects on BBB integrity, depending on which cell type it is removed from [88, 89], it will be important to confirm these antagonistic roles by comparing both knockout strains in the same disease models. Second, define which of the specific β1 integrins expressed at the BBB are responsible for conferring high vascular integrity; do they all contribute or does one e.g.: α3β1 or α6β1 play the major role? Third, determine whether enhanced expression of specific β1 integrins (e.g.: α6β1) can reinforce BBB integrity, and evaluate how this impacts vascular integrity and clinical progression in different animal disease models of hypoxia, ischemia, and inflammatory demyelinating disease. Fourth, define the contribution of the collagen IV-binding integrin α1β1 to vascular integrity, angiogenesis and cellular phenotype. Fifth, determine whether forced expression of the angiogenic α5β1 integrin in remodeling endothelial cells can amplify new vessel growth at the border of an ischemic insult.

## Data Availability

Not applicable.
